# Diode Laser Closure for Cryptoglandular Anal Fistula: A Single-Centre Retrospective Experience

**DOI:** 10.7759/cureus.116939

**Published:** 2026-09-25

**Authors:** Salma Mechhor, Manal Cherkaoui El Malki, Hicham El Bacha, Omeima Cherkaoui El Malki, Nadia Benzzoubeir, Ikram Errabih

**Affiliations:** 1 Gastroenterology and Proctology B Unit, Ibn Sina Teaching Hospital - Mohammed V University in Rabat, Rabat, MAR

**Keywords:** anal fistula, laser closure, recurrence, seton drainage, sphincter preservation

## Abstract

Background

Laser closure of cryptoglandular anal fistula is a sphincter-preserving method with variable success rates across the literature, and the optimal preconditioning of the tract before laser treatment remains undefined. We are reporting here our experience with diode laser closure carried out as part of a systematically staged approach.

Methods

This was a retrospective, descriptive and analytical study, carried out at a single centre and involved 33 patients who were treated in succession from January 2023 to December 2024, with follow-up lasting until August 2026. The laser closure was conducted using a radial fibre with a wavelength of 1470 nm in continuous mode at a power of 10 W, with the fibre being retracted at a speed of 1 mm/s (i.e., 100 J/cm of tract), and without closing the internal opening. In all cases except four patients, preparatory measures, such as seton drainage, abscess drainage or partial opening of the accessory tracts, were carried out before the laser closure. Healing, persistence and recurrence were considered as separate outcomes. Kaplan-Meier estimates were used together with the log-rank test, Fisher's exact test and Mann-Whitney U test.

Results

In 31 out of 33 patients (93.9%), primary healing was achieved, with the median time to healing being 20 weeks. Of these 31 patients who were healed, eight (25.8%) experienced a recurrence - all except one occurring between 9 and 13 months after healing. Sustained healing was found in 23 of 33 patients (69.7%) at a median follow-up of 24 months. None of the patients developed any degree of faecal incontinence. Recurrence was found to be more common in patients with high fistulas than patients with low fistulas (46.2% vs 11.1%; odds ratio 6.86, 95% confidence interval 1.10-42.76; p = 0.043; log-rank p = 0.029). The period between the preparatory procedure and laser closure was shorter in those patients who had a recurrence (median 5.0 vs 12.0 months; p = 0.048), and no recurrences occurred in patients whose tract had been drained for more than eight months.

Conclusions

In the case of a staged approach, laser closure resulted in a high rate of immediate sealing without causing any impairment of continence, and failures occurred in the form of late recurrence rather than persistence. Both the height of the fistula and the duration of drainage before laser closure were associated with recurrence in exploratory analyses. These preliminary findings need to be confirmed by prospective studies and suggest that follow-up should continue well past the first postoperative year.

## Introduction

The estimated number of cases of cryptoglandular anal fistula is between 1.2 and 2.8 per 10,000 people each year, with the condition mainly affecting men between the ages of 30 and 50 years [1]. Although fistulotomy is the most effective treatment, it cuts through the sphincter muscle, and thus the risk of faecal incontinence becomes unacceptable, particularly in cases that are severe or have been operated on more than once. Laser closure of the fistula tract, which was first described in 2011, overcomes this limitation since a radial fibre delivers energy around the inside of the tract, destroying the epithelial lining and sealing it while keeping the thermal damage to only 2 to 3 mm, so that the sphincter complex stays intact even if the procedure fails [2]. A pooled analysis shows a primary healing rate of 67.3% and indicates that continence disturbance occurs in about 1% of patients [3]. The overall figures mask a great deal of variability, since the published success rates vary between 20% and 89% [1]. Throughout the entire procedure, the literature is inconsistent regarding patient selection, the preconditioning of the tract, the laser settings and the management of the internal opening. Preconditioning using a draining seton is in fact one of the only factors shown to affect the outcome [4]. Interpretation is further hampered by the common practice of combining never-healed fistulas with genuine recurrences, and by follow-up periods too short to judge the durability of sealing.

We therefore report our centre's experience with diode laser closure of cryptoglandular anal fistula performed within a systematically staged approach. The primary objective was to determine the rate of sustained healing at last follow-up, defined as healing achieved after laser closure and maintained at the final visit. Secondary objectives were to assess primary healing and time to healing, recurrence after healing (reported separately from primary failure), compare outcomes between first-diagnosed and recurrent fistulas, assess continence and postoperative morbidity, and, as a hypothesis-generating analysis, the factors associated with recurrence.

## Materials and methods

Study design

It is a retrospective, descriptive and analytical study carried out at a single centre and involving patients in consecutive order who received treatment with fistula-tract laser closure (Filac®) from January 2023 up to December 2024, with follow-up extending until August 2026.

Patient selection

Filac® was incorporated into a sphincter-sparing strategy in cases where there was a high risk of impaired continence, specifically, in individuals with recurrent fistula (having previously undergone surgical treatment on one or more occasions - this including abscess drainage, seton placement, fistulotomy or fistulectomy - and in whom the fistula had recurred after healing) or in patients with complex fistulas who had already undergone multiple operative stages. Fistulas of specific origin were excluded when the underlying diagnosis had been established on objective grounds. Crohn's disease was confirmed by ileocolonoscopy with histological examination of biopsies, and anoperineal tuberculosis by histological examination showing caseating granulomas and by microbiological confirmation (direct examination, culture or nucleic acid amplification testing on tissue or pus). Patients with a cryptoglandular fistula did not undergo systematic screening for these conditions; ileocolonoscopy was performed when the history, the digestive symptoms or the local findings raised a suspicion of Crohn's disease.

Patients were eligible if they met either of the two clinical situations described above; Table 1 summarises the full inclusion and exclusion criteria. During the study period, 37 patients were treated, but four were excluded since they had been lost to follow-up or had incomplete records, leaving 33 patients for evaluation.

**Table 1:** Patients selection criteria

Inclusion criteria	Exclusion criteria
Anal fistula of cryptoglandular origin	Fistula related to Crohn's disease
High risk of impaired continence, justifying a sphincter-sparing strategy	Tuberculous fistula
Recurrent fistula, defined as recurrence after healing following one or more previous procedures (abscess drainage, seton placement, fistulotomy or fistulectomy)	Loss to follow-up
Complex fistula already managed through several operative stages	Incomplete medical records

Preoperative assessment

Preoperative assessment consisted of a medical history, an examination of the anal margin, a digital rectal examination, and an anoscopy. Fistulas were classified, according to Parks [5], as extrasphincteric, suprasphincteric, transsphincteric or intersphincteric, on the basis of clinical examination and examination under anaesthesia, supplemented by MRI when available. For the purpose of analysis, fistulas were further dichotomised as high or low: intersphincteric fistulas were classified as low and suprasphincteric fistulas as high, whereas transsphincteric fistulas were classified as high when the tract crossed the upper third of the external sphincter above the puborectalis sling and as low otherwise. A fistula was considered complex when it was high by this definition, when it had more than one tract, when it was anterior in a woman, or when it was associated with a previously drained abscess. Magnetic resonance imaging was not carried out in all patients because of limited availability; it was performed when the fistula was recurrent, when complexity was suspected clinically, or when clinical examination could not identify all the tracts, specifically in order to look for diverticula or accessory tracts.

An abscess was suspected in the presence of perianal pain, swelling, fluctuation or fever, and was confirmed by clinical examination, by MRI when performed, or at examination under anaesthesia.

Definitions

Healing was defined as the closure of both the internal and external openings, with no discharge at two consecutive follow-up visits. It was assessed clinically only.

Failure: Continuous discharge after treatment, with no symptom-free period.

Recurrence: Recurrence was defined as the reappearance of drainage from the previously treated tract, or from its external or internal opening, after complete healing, irrespective of the time elapsed. A fistula arising at a different site, with a separate internal opening, was considered a new fistula rather than a recurrence; no such case occurred in this series.

Sustained healing: Sustained healing was defined as healing achieved after laser closure and maintained without recurrence at the last follow-up visit.

Number of tracts: The number of fistulous tracts found on clinical examination at the time of laser closure, not the number identified before the preparatory procedures.

The preparatory stages involve the surgical steps that come before the laser treatment - whether in the case of a recurrent anal fistula or when dealing with complex anal fistulas - such as draining abscesses and inserting a seton in order to prepare the patients for the laser procedure.

Postoperative discharge was recorded as present or absent, without quantification of volume or duration.

Surgical technique

In the first stage, a draining seton was inserted whenever the tract was not already dry, in order to control the local sepsis and allow the fistula tract to dry out prior to the laser treatment. The seton was left in place until the tract was clinically dry - defined as the absence of purulent discharge on two consecutive outpatient visits, with no fixed minimum duration prescribed. The interval between the last preparatory procedure and laser closure therefore corresponds to the duration of seton drainage. Accessory tracts were defined as any tract arising from the main tract and identified on clinical examination, exploration under anaesthesia or MRI. During the preparatory stage, they were managed by curettage and partial laying open when superficial, or by loose seton drainage when they crossed the sphincter muscle.

The second stage was carried out without bowel preparation, and the procedures were performed under spinal anaesthesia, since this type of anaesthesia causes sphincter relaxation and is useful for controlling pain after surgery. The seton was taken out at the start of the procedure, after which the tract was curetted in order to get rid of any remaining debris. The fistula tract was then catheterised, and the laser fibre was introduced through the external opening by means of the Seldinger technique until the internal opening was reached, thereby ensuring that the laser closure started at the internal opening and continued distally. Accessory tracts that had not closed at the time of laser closure were treated in the same session, each tract being catheterised and lasered separately with identical settings, starting at its internal or junctional opening.

The Leonardo® Dual laser system (biolitec biomedical technology GmbH, Jena, Germany) was used together with a radial fibre which emitted light at a wavelength of 1470 nm and delivered energy around the circumference at 360 degrees. The laser was operated in continuous mode at a power of 10 W, and the fibre was retracted at a constant speed of 1 mm per second over the entire length of the tract, resulting in an energy delivery of 100 J/cm of tract. As the fibre was being retracted, a gentle back-and-forth motion of small amplitude was applied in order to check that the tract had been properly sealed. The sticky feeling felt each time the probe was retracted was regarded as a positive indication of sealing and fibrosis of the tract.

The external opening was then made to coagulate using the laser, and no procedure was carried out to close the internal opening.

All procedures were performed with the same device, the same laser settings and the same staged strategy throughout the study period, with no change in technique over time.

Postoperative care and follow-up

All patients received antibiotic prophylaxis in the form of metronidazole 250 mg taken thrice a day for five days; they were also given a high-fibre diet and sitz baths and asked to take analgesics when needed, and were discharged on the second day after surgery.

Further visits were arranged one week after the procedure and at one, two and six months, as well as at one and two years. Pain was evaluated with the use of the Visual Analogue Scale (VAS) at 6, 12 and 24 hours, on the second day after surgery, and during the follow-up appointments. Continence was checked at each visit both by asking specific questions and by using the Wexner incontinence score [6]. The length of the follow-up period was determined by subtracting the date of laser closure from the date of the last follow-up visit.

Statistical analysis

The analyses were carried out using jamovi (version 2.6), and two-sided tests were applied in all cases, with a p-value of less than 0.05 being regarded as statistically significant. The distribution of continuous variables was assessed using the Shapiro-Wilk test; normally distributed variables are given as the mean ± standard deviation and the others as the median with the interquartile range (IQR). Categorical variables are given as absolute numbers and percentages, and the denominator is included whenever it was different from 33.

The analysis of categorical variables involved the use of Fisher's exact test, with odds ratios given together with their 95% confidence intervals, and for the continuous variables, the Mann-Whitney U test was used.

The time-to-event analyses were carried out using the Kaplan-Meier method, with the groups being compared by means of the log-rank test. The time it took for healing to occur was analysed for the entire cohort and was calculated from the date on which the laser procedure was completed; in the two cases where healing never took place, the patients were censored at the date of their last recorded contact. Survival free of recurrence was assessed from the date of healing and not from the date of the laser treatment, patients without recurrence being censored at the date of their last recorded contact.

Outcomes were additionally compared between patients presenting with a first-diagnosed fistula and those with a recurrent fistula, using Fisher's exact test for healing, recurrence and sustained healing, and the log-rank test for recurrence-free survival. Because recurrent presentation and high fistula height were closely associated in this cohort, these subgroup comparisons were not mutually adjusted.

Given the sample size and the limited number of events, the analysis of the factors associated with recurrence was purely exploratory and was meant for the purpose of forming hypotheses. No adjustment for multiple testing was made, and no multivariable analysis was carried out.

No a priori sample size calculation was performed, since all consecutive eligible patients treated during the inclusion period were included. For information, confirming the differences in recurrence observed between high and low fistulas and between first-diagnosed and recurrent fistulas would require approximately 24 and 38 patients per group, respectively, at 80% power and a two-sided alpha of 0.05, assuming equal group sizes and the proportions observed in this series. Post hoc power was not computed, as it is determined by the observed p-value and adds no information beyond the confidence intervals already reported.

Ethical considerations

The study was carried out in keeping with the Declaration of Helsinki and local institutional ethics requirements. Before it was analysed, the patient data were anonymised, and as a result no identifiable information is included in this report.

## Results

Patients

A total of 33 patients were finally included. Table 2 describes the demographic, radiological and anatomical features of our population. Their mean age was 43.6 ± 11.7 years; 20 patients were men (60.6%), and seven patients were smokers (21.2%). A pelvic MRI was performed in 13 patients (39.4%), and a colonoscopy was performed in four patients (12.1%). The demographic, radiological and anatomical features of the cohort are given in Table 2.

**Table 2:** Baseline characteristics, procedural data and outcomes (n = 33) Data are n (%) unless otherwise specified. Denominators are 33 unless stated. Normality was assessed with the Shapiro–Wilk test; normally distributed variables are reported as mean ± standard deviation and non-normally distributed variables as median (interquartile range). The number of tracts was assessed on clinical examination at the time of laser closure. VAS: visual analogue scale; IQR: interquartile range; MRI: magnetic resonance imaging.

Variable	Value
Patient characteristics	
Age, years — mean ± SD	43.6 ± 11.7
Male sex	20 (60.6)
Smoking	7 (21.2)
Preoperative assessment	
Pelvic MRI	13 (39.4)
Colonoscopy	4 (12.1)
Fistula characteristics	
Intersphincteric	14 (42.4)
Transsphincteric	14 (42.4)
Suprasphincteric	5 (15.2)
Single tract	27 (81.8)
Two tracts	6 (18.2)
Low fistula	19 (57.6)
High fistula	14 (42.4)
Recurrent fistula	17 (51.5)
Posterior location	25 (75.8)
Anterior location	6 (18.2)
Both locations	2 (6.1)
Staged management before laser	
Number of preparatory stages — median (IQR)	2 (1–3)
Laser as a first-line procedure	4 (12.1)
Seton placement	29 (87.9)
Intraoperative abscess drainage	6 (18.2)
Preparation-to-laser interval, months — median (IQR) (n = 29)	8.0 (5.0–13.0)
Postoperative pain, VAS (n = 29)	
6 hours — median (IQR)	3 (3–4)
12 hours — median (IQR)	2 (0–3)
Pain-free (VAS = 0) at 24 hours	15 (45.45)
Pain-free (VAS = 0) at 1 month	25 (86.2)
Complications	
Persistent discharge	10 (30.3)
Local infection	3 (9.1)
Perianal oedema	1 (3.0)
Bleeding, pruritus, incontinence, stenosis	0 (0)
Outcomes	
Primary healing	31 (93.9)
Failure	2 (6.1)
Time to healing, weeks — mean ± SD (n = 31)	19.0 ± 9.0
Return to daily activity, days — median (IQR) (n = 30)	14.5 (10.5–16.0)
Recurrence after healing (n = 31)	8 (25.8)
Time to recurrence, months — median (IQR) (n = 8)	12.0 (10.5–12.2)
Sustained healing at last follow-up	23 (69.7)
Reintervention	10 (30.3)
Repeat laser closure	4 (12.1)
Surgical drainage	6 (18.2)
Total follow-up, months — median (IQR)	24.0 (24.0–28.0)
Follow-up after healing, months — median (IQR) (n = 31)	20.3 (18.5–21.8)

Fistula characteristics

Fourteen fistulas were intersphincteric (42.4%), 14 transsphincteric (42.4%) and 5 suprasphincteric (15.2%); none of the cases involved an extrasphincteric fistula. Fourteen fistulas were classified as high (42.4%) and 19 as low (57.6%), and 17 patients (51.5%) presented with a fistula that had recurred after previous surgery. The tract was posterior in 25 patients (75.8%), anterior in 6 (18.2%) and involved both locations in 2 (6.1%). During the clinical examination, 27 patients (81.8%) were found to have a single remaining tract and 6 (18.2%) had two (Table 2).

Staged surgical strategy

In all but four cases, preparatory measures - such as the insertion of a seton for drainage, drainage of an abscess, or partial opening of the accessory tracts - were carried out before laser closure; in these four cases, the fistulas were simple and had already dried out at the time they were first seen and therefore received laser treatment as the initial procedure. During the laser operation, an abscess cavity was found in six patients and was drained during the same surgical session.

Six patients (18.2%) required drainage of an abscess cavity during the laser session. Primary healing was obtained in four of them, and only one of these four remained healed at last follow-up. Five of these six patients had a recurrent fistula, and four had a high fistula, so this subgroup cannot be considered independently of the two factors described below.

Postoperative course

Pain only occurred during the first hours following the procedure (see Table 2). At 24 hours, 15 patients (45.45%) were entirely free from pain. None of the patients had bleeding, pruritus, anal stenosis, or any degree of faecal incontinence at any time. The only complication that occurred frequently was persistent discharge, which resolved on its own unless it was in patients who were later regarded as having primary failure. Patients were able to resume their normal daily activities after a median period of two weeks.

Healing

In 31 out of 33 patients (93.9%), primary healing was achieved. The cumulative probability of healing was 30.3% at 12 weeks, 57.6% at 20 weeks and 69.7% at 24 weeks; the median time to healing was 20 weeks according to the Kaplan-Meier estimate (the mean being 19.0 ± 9.0 weeks) (Figure 1). The curve reached a plateau of 93.9% at 36 weeks, which corresponds to the last healing event observed in the cohort and not to a predefined assessment time; no further healing occurred thereafter despite continued follow-up. Primary failure occurred in two patients (6.1%), both of whom were managed by salvage drainage.

**Figure 1:**
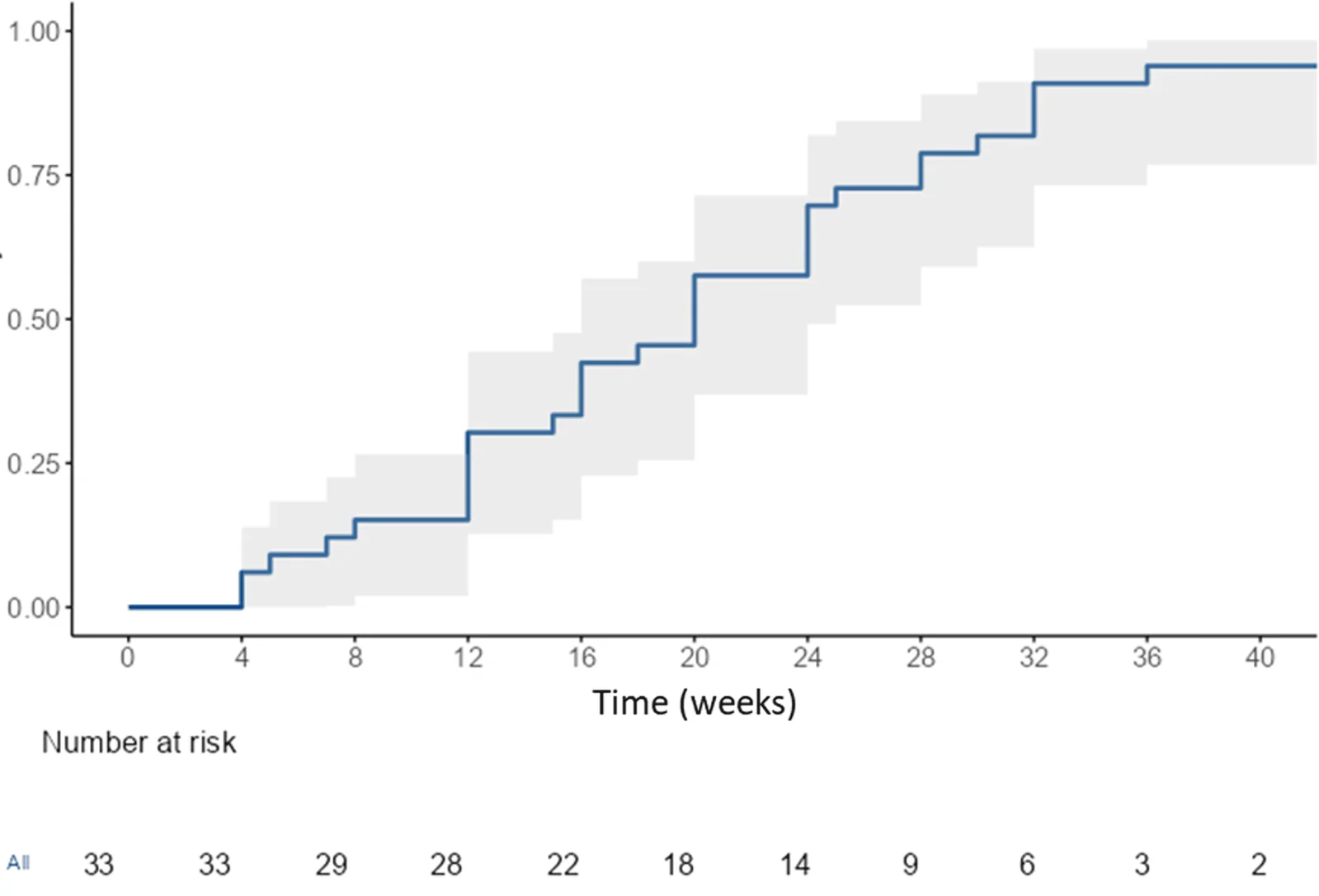
The cumulative probability of fistula healing following diode laser closure (Kaplan-Meier estimate, n = 33) Time is calculated in weeks from the date of laser closure. The shaded area indicates the 95% confidence interval; the number of patients at risk is given below the x-axis. Two patients who never succeeded in achieving healing were censored at the time of their last recorded contact, which explains the plateau below 100%.

Recurrence

Of 31 patients who were healed, eight (25.8%) had a recurrence - all except one occurring between 9 and 13 months after healing. The cumulative incidence of recurrence was 3.2% at 6 months, 19.7% at 12 months and 26.4% at 18 months, and no further recurrences were observed even though the median duration of follow-up after healing was 20.3 months (Figure 2). The median recurrence-free survival could not be reached since less than half of patients suffered a recurrence. Time to healing was longer in patients who subsequently recurred than in those who did not (median 29.0 vs 16.0 weeks; p = 0.016). Four of the recurrences were treated by repeated laser closure and four by surgical drainage. At a median follow-up point of 24 months, sustained healing was noted in 23 out of the 33 patients (69.7%).

**Figure 2:**
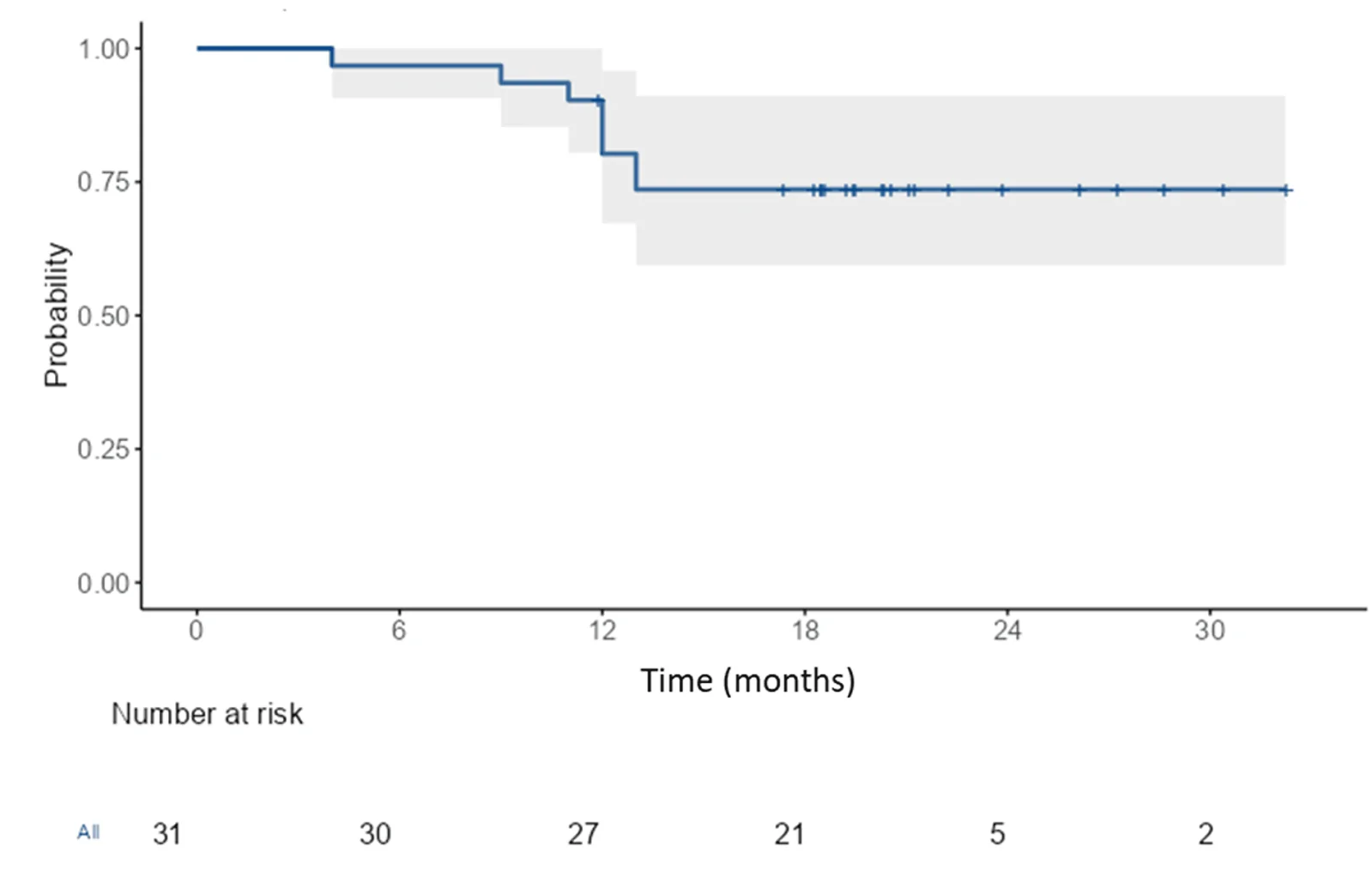
Recurrence-free survival following healing (Kaplan–Meier estimate, n = 31) The time period starts on the day healing occurs. The shaded region shows the 95% confidence interval; tick marks denote censored patients, and the number of patients at risk is given below the x-axis. The two patients who did not achieve primary healing have been excluded. The median recurrence-free survival was not reached.

Outcomes according to first-diagnosed versus recurrent presentation

Sixteen patients presented with a first-diagnosed fistula and 17 with a recurrent fistula (Table 3). The two groups differed at baseline: high fistulas accounted for 13/17 (76.5%) of recurrent cases versus 1/16 (6.3%) of first-diagnosed cases (p < 0.0001), and the median number of preparatory stages was 3 versus 1 (p < 0.001), whereas the preparation-to-laser interval was comparable (8.0 vs 7.0 months; p = 0.72) (Table 3).

**Table 3:** Baseline characteristics and outcomes according to fistula presentation Data are n (%) unless otherwise specified. Recurrent fistula denotes a fistula that had recurred after one or more previous procedures. Denominators for recurrence after healing are the patients who achieved primary healing in each group. P values are from Fisher's exact test, except for the preparation-to-laser interval (Mann–Whitney U test) and for time to healing and recurrence-free survival (log-rank test). All comparisons are unadjusted and exploratory. IQR: interquartile range

Variable	First-diagnosed (n = 16)	Recurrent (n = 17)	p
Baseline characteristics			
High fistula	1 (6.3)	13 (76.5)	<0.0001
Number of preparatory stages — median (IQR)	1 (1–1)	3 (2–4)	<0.001
Preparation-to-laser interval, months — median (IQR)	8.0 (5.0–18.0) (n = 13)	7.0 (5.0–12.3) (n = 16)	0.58
Outcomes			
Primary healing	16 (100)	15 (88.2)	0.48
Time to healing, weeks — median (Kaplan–Meier)	12.0	25.0	0.002
Recurrence after healing	2/16 (12.5)	6/15 (40.0)	0.11
Recurrence-free survival at 18 months, %	86.7	60.0	0.088
Sustained healing at last follow-up	14 (87.5)	9 (52.9)	0.057
Faecal incontinence	0 (0)	0 (0)	—

Primary healing was obtained in 16/16 (100%) first-diagnosed and 15/17 (88.2%) recurrent fistulas (p = 0.48), but healing was significantly slower in recurrent fistulas (median 25.0 vs 12.0 weeks; log-rank p = 0.002). Recurrence after healing occurred in 6/15 (40.0%) recurrent versus 2/16 (12.5%) first-diagnosed fistulas (OR 4.67, 95% CI 0.77-28.41; p = 0.11), with recurrence-free survival at 18 months of 60.0% versus 86.7% (log-rank p = 0.088) (Figure 3). Sustained healing was achieved in 9/17 (52.9%) and 14/16 (87.5%), respectively (p = 0.057). No patient in either group developed faecal incontinence.

**Figure 3:**
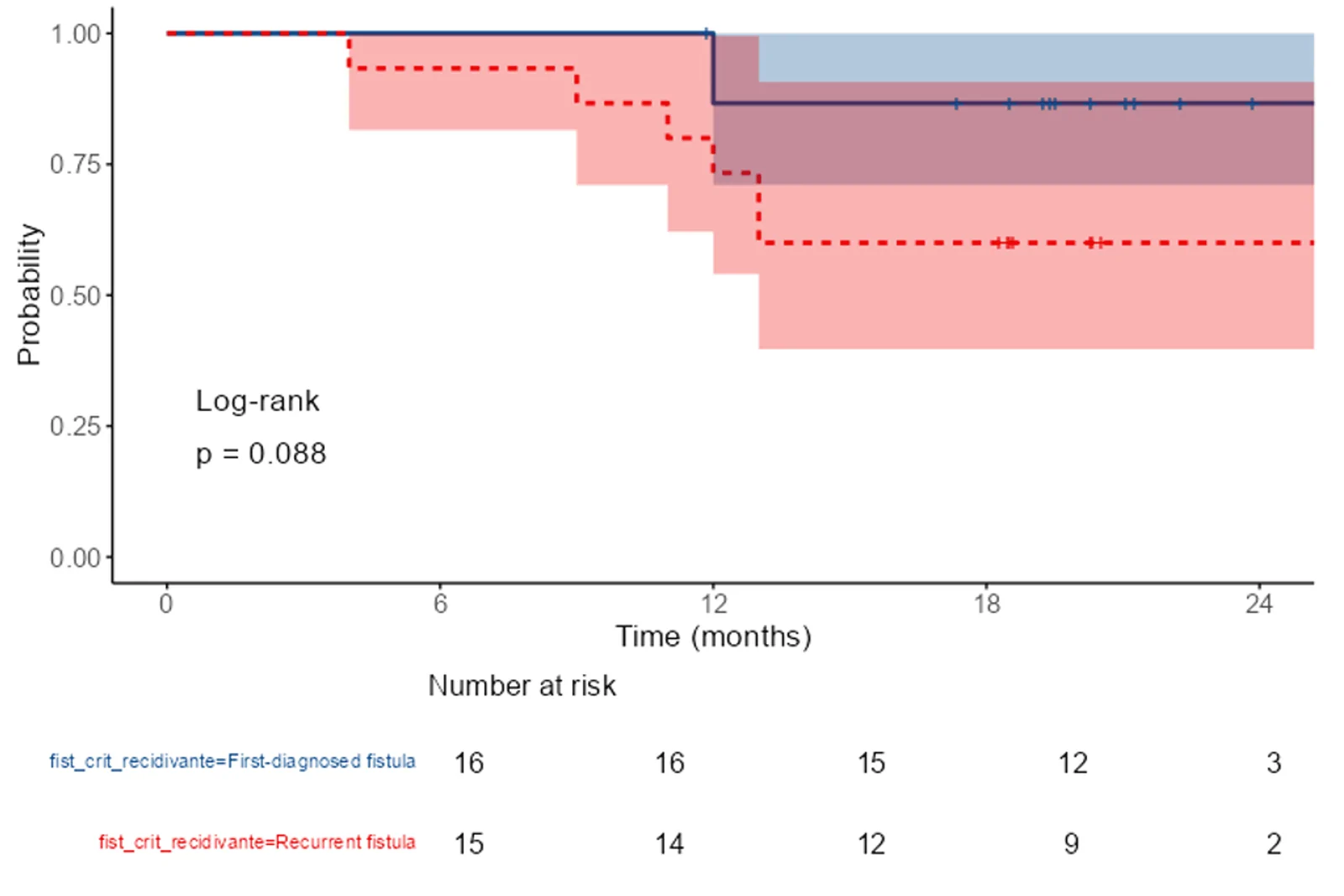
Recurrence-free survival following healing according to fistula presentation (Kaplan-Meier estimates, n = 31) Time is calculated in months from the date of healing. The solid blue line refers to first-diagnosed fistulas (n = 16) and the dashed red line to recurrent fistulas (n = 15); the two patients who never achieved primary healing were excluded. The shaded areas indicate 95% confidence intervals, the tick marks denote censored patients, and the number of patients at risk is given below the x-axis. The log-rank p-value is 0.088, and the median recurrence-free survival was not reached in either group.

Influence of fistula height

Recurrence was much more common in cases with high than in those with low fistulas (46.2% compared with 11.1%; OR 6.86, 95% CI 1.10-42.76; p = 0.043). The recurrence-free survival was likewise shorter in high fistulas (log-rank p = 0.029), the recurrence-free rates being 69.2% and 88.2% at 12 months and 53.8% and 88.2% at 18 months (Figure 4). On the other hand, fistula height did not affect the healing process itself: high fistulas took longer to heal than low fistulas (a median of 25 weeks as compared with 16 weeks), although this difference was not statistically significant (log-rank p = 0.148), and healing was eventually achieved in 13 out of 14 and 18 out of 19 patients, respectively.

**Figure 4:**
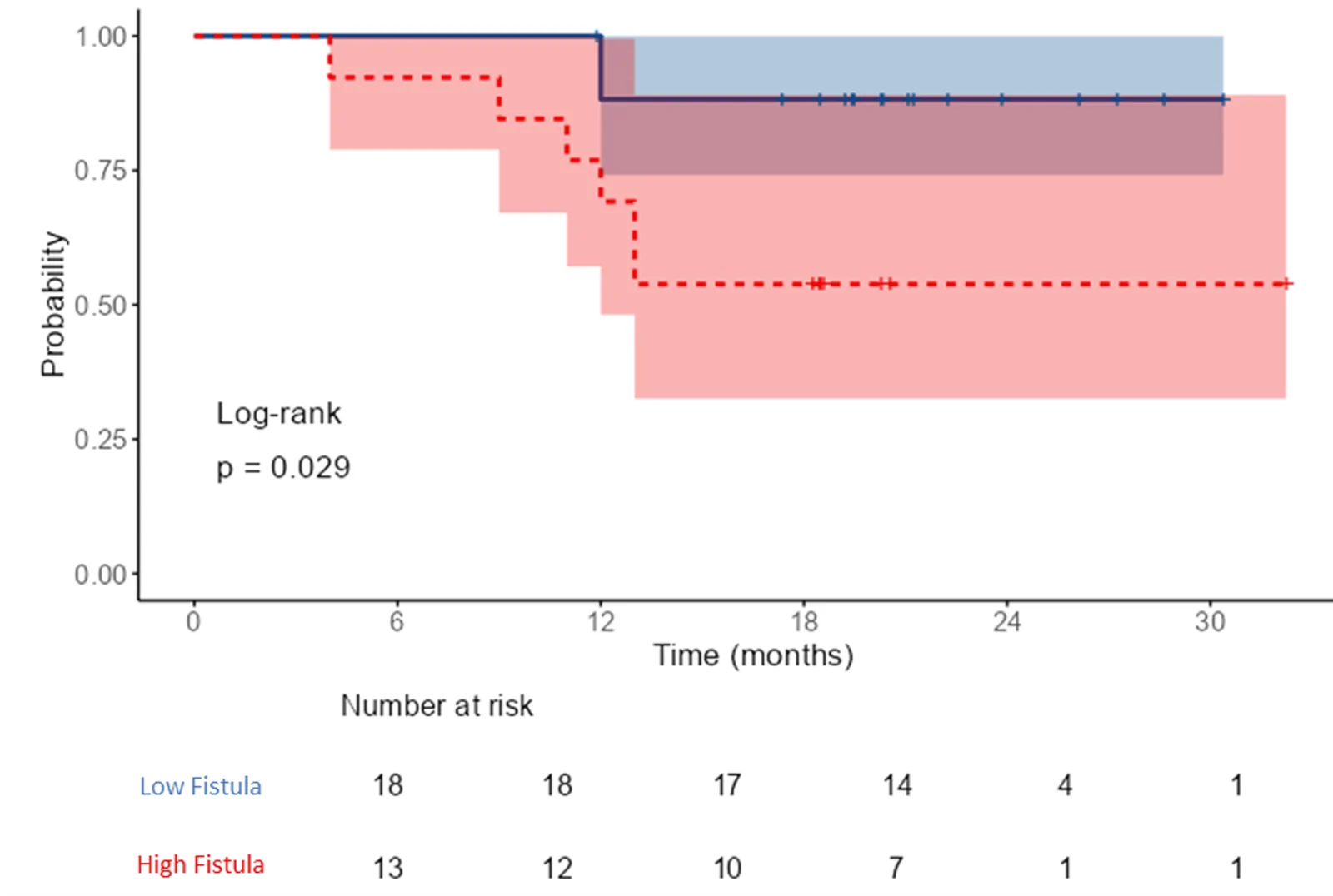
Recurrence-free survival following healing in relation to fistula height (Kaplan-Meier estimates, n = 31) Time is calculated from the date of healing. The solid blue line refers to low fistulas (n = 18) and the dashed red line to high fistulas (n = 13); the two patients who did not achieve primary healing were excluded. The shaded regions show 95% confidence intervals, the tick marks denote censored patients, and the number of patients at risk is given below the x-axis. The log-rank p-value is 0.029.

Influence of the preparation-to-laser interval

In the group of patients who had a recurrence, the interval between preparation and laser treatment was shorter than in those who did not experience a recurrence (median 5.0 months as compared with 12.0 months; Mann-Whitney U = 34.0, p = 0.048). There were no cases of recurrence among patients whose tract had been drained for more than eight months before laser closure. This comparison was univariate and unadjusted, and the eight-month threshold was identified within the present data rather than defined in advance; both findings are therefore exploratory.

## Discussion

In a single-centre study involving 33 consecutive patients who were treated for cryptoglandular anal fistula by diode laser closure, primary healing was obtained in 93.9% of cases, 25.8% of patients who healed experienced a recurrence, and the rate of sustained healing was 69.7% at a median follow-up period of 24 months (IQR 24.0 to 28.0). None of the patients developed faecal incontinence. Two factors were associated with recurrence: fistula height and the interval between the preparatory procedure and laser closure. Both associations were univariate and exploratory.

Rationale and patient selection

The greatest concern in fistula surgery is not failure but postoperative incontinence, and this concern has driven increased use of sphincter-preserving procedures over the last 10 years [7]. The compromise is clearly shown by the largest comparative study currently available: in a group of 3,520 patients, the rates of failure were 28.6% for ligation of the intersphincteric fistula tract, 22.3% for video-assisted anal fistula treatment, 43.9% for laser closure and 25.9% for endoanal flap, but no patient who underwent the latter two procedures experienced a deterioration of continence, whereas continence problems occurred in 1.5% and 7.3% of those who had the first two treatments [8]. A pooled proportional meta-analysis reports a success rate of 63% (95% CI 50-75) and a complication rate of 8% (95% CI 1-18) for laser closure [9]. Laser closure therefore provides a favourable balance even if it does not offer a better cure rate.

The procedure should be indicated in cases where fistulotomy would carry an unacceptable risk of damaging the sphincter, such as when the tract is high transsphincteric, suprasphincteric or extrasphincteric, in the case of anterior fistulas in women, where there is already an impairment of continence, and in patients who have undergone repeated operations [7]. A recent international Delphi consensus reached the same conclusion and considers laser closure a legitimate first-line option in complex disease, while stating that acute cellulitis, undrained collections, active Crohn's disease, and rectovaginal fistula are contraindications [1]. In our practice, we deliberately limited laser closure to these situations, which explains the high number of patients who presented with a fistula that had recurred following previous surgery (51.5%) and the fact that only four patients were treated with it as a first-line procedure. On the other hand, some authors have suggested that their modest results may be explained by the non-selective inclusion of all patients [10]; careful selection, rather than the device itself, seems to be the main factor affecting outcomes.

Preparation of the tract and the timing of laser closure

The institutional approach involves two stages: inserting a seton drain first, then performing laser closure. A loosely placed seton ensures that there is continuous drainage, helps to control local sepsis, allows small secondary tracts to close, and causes the formation of granulation tissue which remodels the lumen and makes the tract more homogeneous and fibrotic - this is exactly the situation in which circumferential laser energy results in uniform shrinkage; it also eliminates the need to identify the internal opening and can be used to guide the fibre across the tract [4,7]. In a series of 175 patients operated on by a single surgeon, the rate of success was considerably higher when a seton or a draining loop was put in during the first stage than when laser closure was carried out directly (70.4% compared with 51.5%; p = 0.0377; odds ratio 0.45), and in that series preconditioning was the only factor that reached statistical significance [4]. The opposite experience is also instructive: a series in which the seton bridge was deliberately omitted found that complete healing occurred in only 42.9% of patients [10]. In our group, 87.9% of patients had a seton, and in all but four cases preparatory procedures were carried out before laser closure; we regard this as a strength of the methodology rather than a confounding factor.

The length of the drainage period enables our data to offer something novel. The interval between preparation and laser treatment was considerably shorter in patients with recurrence than in those without (5 months vs 12 months; p = 0.048), and no recurrences were seen in patients whose tract had been drained for over eight months. This observation is of potential clinical interest since, in contrast to fistula height, the time at which the laser is applied is within the surgeon's control. It should nevertheless be interpreted with caution: the timing of laser closure depended on clinical judgement rather than on a protocol, so the association may equally reflect the characteristics of the fistulas that dried more slowly, and the eight-month threshold was identified within the present data rather than defined in advance. This finding is therefore hypothesis-generating and does not establish a causal effect of drainage duration. Reported drainage periods vary widely, ranging from a median of 10 to 14 weeks [4,11] to a median of 32 weeks [7], while expert consensus specifies only a minimum of two months and leaves the timing to clinical judgement and imaging [1]. Our results are compatible with the longer part of that range.

Technical protocol and the internal opening

Every current series makes use of a diode laser with a wavelength of 1470 nm [12-14]; the absorption peak at this wavelength in water results in effective tissue shrinkage while limiting the area of thermal damage to about 2 to 3 mm around the tract [3,10], and it is now universally agreed [1]. Although power settings remain debated, we must justify our choice of 10 W, since consensus suggests 12 W [1]. We believe that power alone is a poor way of describing the dosage and that linear energy density is the parameter which has a physical significance; on this basis, our value of 100 J/cm at a withdrawal speed of 1 mm/s is close to the approximately 120 J/cm reported in the largest published series and higher than 39 and 90 J/cm values previously reported [11,13,15]. So our procedure lies within rather than below the effective range. This agrees with the meta-regression of the combined literature, which found no significant relationship between technical parameters and success rates [9].

We do not close the internal opening, which is the point at which our practice most clearly differs from the consensus recommendation [1]. That said, the comparative data do not resolve the issue since no significant difference in healing was observed between the procedures carried out with or without closure (66.7% compared with 50%, p = 0.34) [7]; the pooled analysis also failed to show any advantage [3], and in the univariate analysis of a group of 175 patients, closure was not a significant predictor (74.1% vs 66.2%; p = 0.42) [4]. The most direct test of the hypothesis was a study in which a single team introduced purse-string suturing into their standard technique after having previously treated a cohort without closure: complete healing was 42.9% in the group that had the suturing and 40% in those who did not, so the authors concluded that primary closure has no positive impact on healing [10]. The only remaining argument in favour of closing the opening is the presence of a wide orifice, since a failure rate of 84.6% has been reported when the internal opening is wide as opposed to 50.6% when it is narrow [16]. Our primary healing rate of 93.9% indicates that omitting closure does not affect sealing provided the tract is properly prepared and the orifice has had the opportunity to remodel around a long-standing seton.

Prognostic factors

The factors most frequently cited as being adverse are undetected secondary tracts and a broad fistula lumen, since both of these prevent the fibre from sticking to the lining epithelium [3]; it is believed that the shrinking effect causes tracts with a diameter of 4 to 5 mm or less to close, and this provides yet another reason for extending the drainage period, because the tract adjusts its diameter to that of the drain [1,4]. Tract length has also been suggested as a factor, with shorter fistulas showing better healing in one of the series [17]. However, fistula height is more disputed, as it has been described both as a factor leading to failure [3,15] and as having no predictive value [4].

Our findings align with this pessimistic trend. Recurrence was much more common in cases with high than in those with low fistulas (46.2% compared with 11.1%; OR 6.86, 95% CI 1.10-42.76; p = 0.043), and recurrence-free survival was correspondingly shorter (log-rank p = 0.029). The wide confidence interval reflects the small number of events (n = 8) and therefore prevents a precise estimate of the effect size; the association should be interpreted as hypothesis-generating. It is important to note that having a high fistula did not interfere with the healing process itself, although high fistulas took longer to heal (median 25 weeks as compared with 16 weeks). This difference was not statistically significant, and healing was eventually achieved in 13 of 14 and in 18 of 19 patients with low fistulas. Thus, fistula height seems to affect the durability of closure rather than the initial success of healing - a point that may help explain the conflicting results published elsewhere, since studies that reported only primary healing would not have been able to detect this.

Persistence versus recurrence

The comparison between the series is difficult because definitions of healing and failure differ [18], and some researchers have combined non-healing and recurrence into a single category [3]. We deliberately kept the two outcomes separate because they have different clinical significance and different implications for treatment. It is important to make this distinction since the main way in which failure occurs after laser closure is by persistence rather than by true recurrence: In a systematic review of 158 cases of failure, 97% represented the absence of primary healing and only 3% were cases of real recurrence [3], and the same trend was observed in a cohort from a tertiary centre (81% vs 19%) [7] and in a series of 175 patients (21.7% persistence compared with 11.4% recurrence) [4]. Our series is unusual in that primary healing was achieved in 93.9% of patients, meaning almost all failures were late recurrences occurring between 9 and 13 months after healing in all but one instance. We think this pattern reflects the systematic, staged preparation of the tract, which specifically addresses the factors that cause early non-healing; as a result, the clinical issue shifts from initial sealing to the long-term durability of that seal. Consistent with this, patients who recurred took longer to heal initially, which suggests that slow healing and late recurrence may reflect the same underlying tract characteristics rather than two distinct modes of failure. It also means that follow-up should continue well beyond the first postoperative year, in accordance with the schedule recommended by consensus [1].

Strengths and limitations

The strengths of this study lie in the homogeneity of the cohort and of the treatment. All consecutive eligible patients treated over a three-year period were included; all procedures were performed by the same team with the same device and identical settings within a uniformly staged strategy; and follow-up was prolonged (median 24 months, up to 38), which is uncommon in this literature, and allowed late failures to be captured. Persistence and recurrence were assessed as distinct outcomes with predefined definitions, and time-to-event methods were used rather than crude proportions at an arbitrary time point.

This study has two principal limitations: it is a retrospective series of 33 patients, which limits the precision of all estimates and precludes multivariable adjustment, and it has no comparator group, so no claim of superiority over other sphincter-sparing techniques can be made. These limitations are shared by the existing evidence on laser closure, which is almost entirely based on retrospective, single-center cohort studies using inconsistent definitions and variable periods of follow-up [8], and which has been rated as having a low or very low level of evidence despite efforts to standardize it [1].

Four further limitations apply, which are as follows:

First, of the 37 patients treated during the study period, four (10.8%) were excluded because they were lost to follow-up or had incomplete records; their outcomes are unknown, and the direction of any resulting bias cannot be determined.

Second, magnetic resonance imaging was carried out in only 39.4% of patients, owing to its limited availability, and only in complex or recurrent fistulas or when clinical examination could not identify all the tracts; occult accessory tracts can therefore not be formally ruled out, and healing was likewise assessed clinically, without imaging confirmation, which may overestimate true anatomical closure.

Third, the number of recurrence events was small (n = 8), so the associations reported are exploratory and unadjusted, with no correction for multiple testing. This is compounded by the collinearity between recurrent presentation and high fistula height (76.5% vs 6.3%), a direct consequence of our indication policy: their respective contributions to recurrence cannot be disentangled with so few events, and the higher recurrence rate observed in previously operated fistulas did not reach significance and should not be interpreted as an independent effect.

Fourth, the timing of laser closure was determined by clinical judgement rather than by protocol, so the association between a longer drainage interval and the absence of recurrence may be partly confounded by indication; investigations for Crohn's disease and tuberculosis were similarly driven by clinical suspicion, so an occult specific cause cannot be formally excluded in every patient.

## Conclusions

The use of laser treatment for cryptoglandular anal fistulae has shown to be both safe and effective at our centre, without causing any functional impairment in a group that is at a high risk of incontinence. Our experience mainly indicates that the key aspect of the operation occurs before the laser is turned on: a tract which has been drained for a sufficient period and has therefore become dry and fibrotic is more likely to remain closed, whereas the height of the fistula, which cannot be altered, mainly influences how long the closure will last. In this situation, failure is therefore less a matter of achieving a good seal than of maintaining it over time, and the follow-up needed to identify such failures is longer than what is usually provided in published series. These findings, based on a small retrospective study, present a hypothesis that needs to be tested rather than a rule to apply.
